# Mothers’ and fathers’ mind‐mindedness influences physiological emotion regulation of infants across the first year of life

**DOI:** 10.1111/desc.12689

**Published:** 2018-06-19

**Authors:** Moniek A.J. Zeegers, Wieke de Vente, Milica Nikolić, Mirjana Majdandžić, Susan M. Bögels, Cristina Colonnesi

**Affiliations:** ^1^ Research Institute of Child Development and Education University of Amsterdam Amsterdam The Netherlands

## Abstract

The main aim of this study was to test whether mothers’ (*n* = 116) and fathers’ (*n* = 116) mind‐mindedness predicts infants’ physiological emotion regulation (heart rate variability; HRV) across the first year of life. Three hypotheses were examined: (a) parents’ mind‐mindedness at 4 and 12 months predicts infants’ HRV at 12 months over and above infants’ initial HRV levels at 4 months, (b) mothers’ and fathers’ mind‐mindedness independently predict infant HRV, and (c) the effects of mind‐mindedness on infant HRV (partially) operate via parenting behaviour. Infants’ HRV was assessed during rest and a stranger approach. Mind‐mindedness was assessed by calculating the proportions of appropriate and non‐attuned mind‐related comments during free‐play interactions, and parenting quality was observed at 4 and 12 months in the same interactions. Path analyses showed that mothers’ appropriate mind‐related comments at 4 and 12 months predicted higher baseline HRV at 12 months, whereas mothers’ non‐attuned comments predicted lower baseline HRV at 12 months. Similar, but concurrent, relations were found for fathers’ appropriate and non‐attuned mind‐related comments and infant baseline HRV at 12 months. In addition, fathers’ appropriate mind‐related comments showed an indirect association with infant baseline HRV at 12 months via fathers’ parenting quality. With regard to infant HRV reactivity during the stranger approach, mothers’ appropriate mind‐related comments at 4 months and fathers’ non‐attuned mind‐related comments at 12 months predicted a larger HRV decline during the stranger approach at 12 months. Infants’ HRV at 4 months did not predict parents’ later mind‐mindedness. The results indicate that mothers’ and fathers’ appropriate and non‐attuned mind‐related speech uniquely impacts the development of infants’ physiological emotion regulation.

**RESEARCH HIGHLIGHTS**

- This study examined the relations between parents’ mind‐mindedness and infants’ physiological emotion regulation capacity throughout the first year of life.
- Mothers’ and fathers’ appropriate and non‐attuned mind‐related speech independently predicted infants’ emotion regulation (Heart Rate Variability; HRV) at 12 months.
- Maternal mind‐mindedness at 4 and 12 months predicted infants’ HRV at 12 months, while paternal mind‐mindedness at 12 months concurrently associated with infants’ level of HRV.
- Mind‐mindedness of fathers showed a direct and indirect effect (via parenting quality) on the infant's emotion regulation capacity at 12 months.

## 1 INTRODUCTION

Most parents want their child to have emotions, but not be overpowered by them; to feel disappointed but not to cave in; to be thrilled but not get hysterical, to feel some anxiety (and therefore be careful), but not become overly worried or avoiding. In other words, parents hope that their child will find a balanced way to deal with the impact of emotions—to be able to regulate their emotions. For a substantial period of childhood—the first years—the child's regulation of emotions crucially depends on the parent's ability to manage the physical and emotional states of the child (Taipale, 2016). As infants possess only a limited set of self‐regulating behaviours, the parent's daily interpretations of and responses to the infant's arousal form the infant's first experiences with the onset and attenuation of emotional states. This leads to the question if and what characteristics of the early parent–child relationship predict the development of children's emotion regulation capacity.

In behavioural science, emotion regulation refers to processes that affect the impact and/or time of an emotional response, either intentional or spontaneous, occurring at the physiological, behavioural, and cognitive levels (Gross, 2013). Although emotion regulation is a product of the interplay between various domains, it is known to be greatly affected by the flexibility of the body to up‐ or downregulate emotional arousal on a momentary basis (Appelhans & Luecken, 2006; Gross, 1998). A commonly used index to assess physiological regulation is high‐frequency (HF) heart rate variability (HRV; Task Force of the European Society of Cardiology and the North American Society of Pacing and Electrophysiology, 1996). The HF HRV measure is a noninvasive index of the variation in duration between subsequent heart beats, and provides information on the degree to which the parasympathetic nervous system (PNS) influences heart functioning (Porges, 2001).

The PNS is part of the autonomic nervous system and promotes functions associated with the restoration and conservation of bodily energy and the resting of vital organs. Activation of this system typically results in inhibitory effects on body functions, such as slowing down the heart rate (Porges, 2011). The other branch of the autonomic nervous system, the sympathetic nervous system (SNS), prepares the body for an adaptive response to external challenges. Activation of the SNS results in, for instance, accelerated heart rate. The PNS and SNS thus have opposite effects on the body and continuously interact with each other in maintaining physiological adaptation to the environment (Pumprla, Howorka, Groves, Chester, & Nolan, 2002).

Heart rate variability is typically assessed during a resting (baseline) period or during an active situation, reflecting two different aspects of emotion regulation. The level of HRV during rest (i.e., baseline) is considered to represent an individual's general capacity and flexibility to regulate emotions (Appelhans & Luecken, 2006). A high baseline HRV level, indexing higher PNS activity, represents more regulatory capacity (Propper & Moore, 2006). For example, high baseline HRV allows the autonomic nervous system to flexibly adapt to environmental changes, as it can more rapidly withdraw parasympathetic activity. As far as emotions are concerned, more variation in PNS activity is associated with an increased capacity to regulate rapid shifts between high and low arousal states (Appelhans & Luecken, 2006).

A body of empirical work underlines baseline HRV as a physiological marker of emotion regulation capacity in children. In toddlers and older children, high baseline HRV has been proven to relate to, among other things, greater attention control, social competence, empathy, regulation of distress during frustrating events, and lower levels of aggression (Beauchaine, 2015). However, in early infancy, particularly in the first 6 months of infant development, the role of baseline HRV as an index of regulatory processes is less clear. Higher levels of baseline HRV in this developmental phase have been linked to positive affective expressions; for example, during interactions with strangers, sustained visual attention and processing speed, stronger responsiveness to novel stimuli, but also to more negative reactivity (crying, negative emotionality; e.g., Beauchaine, 2001; Fox, Schmidt, & Henderson, 2000; Propper & Moore, 2006). Therefore, high baseline HRV in early infancy has been proposed to represent, next to emotion regulation capacity, the infant's tendency to actively engage with the environment, and a certain responsiveness, both positive and negative, to environmental challenges (Beauchaine, 2001; Fox, 1989).

Mean levels of baseline HRV typically increase with age from infancy through middle childhood, suggesting that the PNS matures in early childhood (e.g., Bar‐Haim, Marshall, & Fox, 2000; Bornstein & Suess, 2000; Izard et al., 1991; Patriquin, Lorenzi, Scarpa, Calkins, & Bell, 2015; Stifter, Fox, & Porges, 1989). This increase is in line with the vast expansion of behavioural emotion regulation strategies in the first years due to improved motor, communication and cognitive skills (Sroufe, 1997). However, there are a few studies in which infants’ HRV showed no increase over time (e.g., Fracasso & Porges, 1994; Snidman, Kagan, Riordan, & Shannon, 1995). The individual stability of baseline HRV over time also showed mixed results: several studies have found no stability over the first year of life (e.g., Porter, Bryan, & Hsu, 1995; Stifter et al., 1989), yet other studies have reported moderately stable HRV levels from 3 to 36 months (DiPietro, Bornstein, Hahn, Costigan, & Achy‐Brou, 2007; Izard et al., 1991; Patriquin et al., 2015; Porter et al., 1995). These mixed outcomes strongly suggest that there are large individual differences in the development of the autonomic nervous system.

Levels of HRV during active situations, for instance, situations that involve social interactions, are indicative of how the body regulates affective arousal during these particular situations (Porges, 2011). Emotion regulation during socially stressful situations is reflected by a decrease in HRV relative to baseline (Porges, 2003; Shahrestani, Stewart, Quintana, Hickie, & Guastella, 2014). When the PNS withdraws its inhibitory influence on the heart, activity of the SNS is allowed to become more prominent, enabling active coping (e.g., a fight or flight response). A decrease in HRV during stressful situations thus reflects withdrawal of the PNS and active coping with the external challenge. It is expected that a higher general regulatory capacity (as reflected in *higher* HRV during resting state) is associated with more emotion regulation during a stressful or challenging situation (Porges, 2011), which is reflected in a stronger HRV *decline* during the challenge. Most empirical studies support the notion that stronger HRV decline during stressful or challenging situations reflects active emotion regulation in children. A recent meta‐analysis synthesized studies on children's (0 to 12 years) HRV change during social tasks (Shahrestani et al., 2014). The results suggest that typically developing children show HRV decreases from baseline during socially stressful situations, such as in the strange situation procedure or the still face paradigm.

### 1.1 Predicting infant emotion regulation: parents’ mind‐mindedness

As mentioned above, in infancy the autonomous nervous system is not fully developed and is maturing (Bornstein & Suess, 2000). Moreover, infants are not able to adjust the environment to fit their emotional states; they display some but limited behavioural strategies to deal with emotions, such as turning away from stimuli that cause overarousal, sleeping, self‐distraction (e.g., playing with a toy), and self‐soothing behaviours (e.g., sucking). The regulation of their daily emotion thus largely depends on other individuals, most often the parents. Parents evaluate their infant's cues and decide when to feed and comfort their infant, when to keep it warm and protect it against overarousing stimuli. It has therefore been proposed that parents not only act as crucial modulators of the infant's states, but they are also, to some extent, in charge of the infant's moment‐to‐moment states (Taipale, 2016).

Many empirical studies on the contribution of environmental factors to the development of emotion regulation in infants have examined the influence of the infant–parent attachment relationship (e.g., Haley & Stansbury, 2003; Hill‐Soderlund et al., 2008; Perry, Calkins, & Bell, 2015). According to attachment theory, infant–parent relationships are embedded within a dyadic mutually regulating system that affects or even partially constitutes the infant's later capacity for emotional regulation (Bowlby, 1969/1982). This theory proposed that newborn infants are biologically predisposed to maintain proximity to a caregiver in order to increase the chance of protection and survival. Everyday interactions with the caregiver gradually determine infants’ anticipations of how caregivers react to their expression of distress. These expectations are thought to be stored in a flexible cognitive framework (referred to as internal working models; Bowlby, 1973). It is suggested that the securely attached infant has constructed a general expectation that his or her emotional cues will be responded to by the parent (Ainsworth, Bell, & Stayton, 1974; Cassidy, 1994). The expression of negative affect (e.g., discomfort, anger, fear) has thus become associated with the parent's helpful response. The securely attached infant may therefore experience negative affect for a short time, trusting that arousal in the company of the parent will not lead to disruption beyond his or her coping abilities (Fonagy, Gergely, Jurist, & Target, 2004). The infant–parent system thus serves as a context in which the secure infant develops a sense of efficacy in modulating affect.

Attachment seems to provide an important framework for understanding the onset of emotion regulation. A question that follows is: how do infants come to perceive their parents as being sufficiently fine‐tuned to their emotion, thereby building trust in the regulatory function of the infant–parent system? A concept that has often been put forward as a key facilitator of attachment and socio‐emotional development is parents’ mind‐mindedness (Meins, 1997; Meins, Fernyhough, Fradley, & Tuckey, 2001; Zeegers, Colonnesi, Stams, & Meins, 2017). Mind‐mindedness is defined as parents’ tendency to treat their infant as a mental agent, and is assessed during infancy as parents’ tendency to comment appropriately or in a non‐attuned manner on their infant's putative internal states during free‐play situations (Meins, 1997; Meins et al., 2001). The appropriate and non‐attuned indices reflect two orthogonal dimensions of mind‐mindedness, unrelated to each other (Meins et al., 2003, 2012). Appropriate mind‐related comments indicate attunement to and validation of the infant's internal state. Non‐attuned comments reflect the extent to which misinterpretations of the infant's state emerge as a result of parents projecting their own state of mind or imposing their own agenda on the infant (Meins, 2013). Greater mind‐mindedness is indexed by high levels of appropriate mind‐related comments or low levels of non‐attuned mind‐related comments.

Mind‐mindedness is a construct at the interface of representational and behavioural assessment of the infant–parent relationship: parents’ mind‐related speech reflects the mental tendency to form theories of the infant's mind (i.e., to mentalize) as well as the behavioural tendency to explicitly (verbally) communicate theories of the infant's mind during interactions (Meins, 2013; Meins et al., 2012). On the one hand, the behavioural component of mind‐mindedness (mind‐related speech) may have a direct effect on the organization of the infant's affect. An important developmental step during infancy involves the infant's increasing ability to categorize natural and artificial things (e.g., animal types, furniture), which starts to develop by 3 to 4 months of age (Behl‐Chada, 1996; Eimas & Miller, 1992; Quinn & Eimas, 1996). Growing research suggests that spoken words help infants to categorize already from the age of 9 months (for a more elaborate review on the role of language in emotion, see Lindquist, MacCormack, & Shablack, 2015). For example, 9‐month‐old infants use words (and not tones, sounds or facial expressions) as cues for understanding which objects in the world are similar and distinct (Dewar & Xu, 2009; Xu, 2002). Similarly, it has been proposed that emotion labelling may be an important cue for helping infants and young children appreciate emotion categories and apply those categories to their own experiences and observations (Lindquist et al., 2015). This theory indicates that early mind‐related speech may impact the infant's developing sense of emotion recognition and organization, and therefore may exert a direct influence on the infant's emotion regulation capacity across the first year of life.

On the other hand, the mental component of mind‐mindedness (mentalizing) may enable parents to respond to and interact with their infant in an attuned manner, both on a conscious and an unconscious level (e.g., by being sensitively responsive to infant cues, synchronous, cooperative, warm, and autonomy granting; Camoirano, 2017; Zeegers et al., 2017). The importance of parents’ “mind‐minded” stance on infants’ developing emotion regulation may become more evident in an example of the parent and infant interacting in a peekaboo game. If, during the game, the infant signals that he or she is over‐stimulated (e.g., by turning away from the caregiver, tuning out, frantic movements, etc.), the parent's appropriate understanding of the behavioural signal in terms of internal states (e.g., feeling overwhelmed) is critical in guiding the parent's reaction. When the parent accurately understands the signal, the parent is more likely to show an attuned response: pause the game, enabling the infant's autonomic system to recover from the heightened arousal. When these types of interactions occur on a regular basis, the infant's autonomic nervous system practises with shifting between different states, and maintaining physiological homeostasis. The accumulation of these interactions may then support the flexible working of the infant's autonomic system (Moore et al., 2009).

Conversely, when the parent does not have a mind‐minded stance, he or she may not see or misinterpret the infant's signal and is more likely to continue the peekaboo game. The infant then does not experience recovery from heightened physiological arousal in a way that is matched to his or her current state of being. If such interactions accumulate over time, the autonomic nervous system may adapt to an over‐stimulating environment by maintaining lower overall parasympathetic activity (lower baseline HRV). Hence, parents who are able to appropriately interpret their infant's state of mind seem better able to adjust themselves and the environment in a way that allows the infant to regulate arousal.

This example suggests that mind‐mindedness may be a key predictor of infants’ developing emotion regulation capacity, and that the effect of mind‐mindedness may operate through parenting behaviour. This notion is supported by a recent meta‐analysis which showed that mind‐mindedness (together with two other measures of parental mentalization: reflective functioning and insightfulness) predicted infant attachment security directly, but also indirectly via parents’ sensitive responsiveness (Zeegers et al., 2017). Next to predicting infant attachment security, parental mind‐mindedness has frequently been shown to predict children's own mentalizing abilities at the ages of 2 to 5 years, such as false belief understanding, and the understanding of discrepant desires and visual perspective taking (e.g., Laranjo, Bernier, Meins, & Carlson, 2010; Lundy, 2013; Meins et al., 2002, 2003, 2013). Children with mind‐minded parents thus find it easier to interpret other people's behaviour within a mentalistic framework. Children's mentalizing abilities have been argued to be an important component of emotion regulation, particularly in the context of social relationships (e.g., Sharp et al., 2011). In other words, there is a body of work suggesting that parents’ use of appropriate mental state language before infants can speak impacts the child's later socio‐cognitive skills relevant to emotion regulation.

### 1.2 The present study

In the present study we aimed to complement existing literature on the development of infants’ emotion regulation capacity. We examined three hypotheses concerning the relations between mothers’ and fathers’ mind‐mindedness and infants’ physiological emotion regulation. First of all, we hypothesized that parents’ mind‐mindedness at 4 and 12 months would predict infants’ HRV levels at 12 months, over and above infants’ initial HRV levels at 4 months. We expected that appropriate mind‐related comments would predict higher baseline HRV (positive association) and a stronger decline in HRV levels (negative association) during a socially challenging task involving a male stranger at 12 months, taking into account infants’ HRV at 4 months. Conversely, we expected that parents’ non‐attuned mind‐related comments would predict lower levels of baseline HRV and less HRV decline during the stranger situation at 12 months.

There is little research on the determinants of parents’ mind‐mindedness. The studies that are available suggest that the tendency to be mind‐minded is dependent on the familiarity with the interaction partner and therefore is an indicator of people's relationship quality (Meins, Fernyhough, & Harris‐Waller, 2014). Since relationships are an accumulation of a history of interactions between individuals, mind‐mindedness may be influenced by how an infant expresses and responds to arousal from birth. However, previous research has shown that mind‐mindedness is unrelated to infant temperament, suggesting that individual infant characteristics do not determine parents’ mind‐mindedness (Meins, Fernyhough, Arnott, Turner, & Leekam, 2011). The results of this study furthermore highlight that caregiver mind‐mindedness already starts to take shape during pregnancy, and therefore primarily taps into parents’ representations of their children, rather than the child's behaviour in itself. These results suggest that parents’ mind‐mindedness is not affected by the infant's capacity to express and regulate emotion. Because further evidence on the relation is lacking, we simultaneously examined whether infant HRV predicts mind‐mindedness (i.e., we tested the transactional relations between infant and parent measures).

Second, we hypothesized that fathers’ and mothers’ mind‐mindedness would independently predict the child's emotion regulation. It is only 15 years since fathers have been included more systematically in studies on parenting and young children's development in general, and emotion regulation specifically. Fathers’ interaction style with their children is characterized as being generally different from mothers’ interaction style: more lively, excitatory, unpredictable, emotionally arousing. Fathers’ interactions may have a unique effect on children's development and mental health (Bögels & Perotti, 2011; Lamb, 2000; Paquette, 2004). Especially because paternal play may induce high arousal, fathers’ interpretations of, and responses to, their infant's emotional expressions may be relevant in understanding how infants internalize strategies to deal constructively with highly emotionally charged situations (Lamb, 2000; Martins, Soares, Martins, & Osório, 2016).

While a body of research exists on the impact of mothers’ mind‐mindedness on children's emotional development, research on the impact of fathers’ mind‐mindedness is to our knowledge limited to three studies on the prediction of infant attachment security and the prediction of children's social understanding and self‐regulated conduct (Arnott & Meins, 2007; Gagné, Bernier, & McMahon, 2018; Lundy, 2003, 2013). Although these studies had small sample sizes, the outcomes suggest that fathers’ mind‐mindedness is also predictive of children's socio‐emotional development, highlighting the relevance of including both mothers and fathers in developmental research on mind‐mindedness. In the present study we expected that mothers’ and fathers’ mind‐mindedness at 4 and 12 months would independently predict infant baseline HRV and HRV decline at 12 months.

Third, we hypothesized that mind‐mindedness predicts infant HRV via parenting behaviour. As outlined earlier, mind‐mindedness is a construct at the interface of behavioural and representational/mental abilities. Mind‐related speech may directly affect children's emotion regulation. However, the parents’ proclivity to take the infant's perspective cannot be measured directly and may exert its effect on emotion regulation via parenting behaviour. We assessed parenting behaviour in terms of parents’ responsiveness, intrusiveness, warmth/affectivity and negativity during parent–infant interactions at 4 and 12 months. We expected that parents high in mind‐mindedness would show a higher quality of parenting (i.e., high in responsivity and warmth, low in intrusiveness and negativity) during interactions at 4 and 12 months, which, in turn, would positively affect infant regulation at 12 months. We further expected that the direct effect of mind‐mindedness on infant HRV would hold over and above the indirect effect via parenting quality.

## 2 METHODS

### 2.1 Participants

Families from the present study participated in an ongoing longitudinal study on social development from infancy to middle childhood (de Vente, Majdandžić, Colonnesi, & Bögels, 2011). Couples expecting their first child were recruited through advertisements in magazines and flyers distributed by midwives. Approval from the ethical committee was obtained, and written informed consent was obtained from all participants. Families were excluded if the infant's birth weight was under 2500 g, or if the infant had neurological disorders, or an APGAR score below 8. Families received a gift voucher after every measurement. We analysed two measurement waves in the current study: 4 months (Time 1) and 12 months (Time 2). At Time 1, when the infant was 4 months old (*M*_age_ = 4.2, *SD* = 0.33), 135 fathers and mothers and their firstborns participated (75 girls, 55.6%). At Time 2, when the child was 12 months old (*M* = 12.4, *SD* = .72), 131 fathers (3% missing) and 130 mothers (4% missing) took part (*M* = 12.4, *SD* = .72). Attrition was due to couples indicating that they did not have enough time to participate in the longitudinal study. Mean age of mothers at Time 1 was 31.30 years (*SD* = 4.41) and 33.87 years (*SD* = 4.77) for fathers. The mean educational level of parents was fairly high, *M* = 7.10, *SD* = 1.25 for mothers, and *M* = 6.53, *SD* = 1.55 for fathers (on a scale from 1: primary education to 8: university degree). Parents were primarily Caucasian. All parents were living together at the time of the assessments.

On an average weekday (Monday to Friday) at 4 months, 40% of the infants were cared for by their mother, 11% were cared for by their father, and 49% were in non‐parental care. On an average weekday at 12 months (Monday to Friday), 34% of the infants were cared for by their mother, 11% were cared for by their father, and 55% were in non‐parental care. Fathers never took care of their child more than 2 days of the (work)week at 4 and 12 months. On an average weekend day at both 4 and 12 months, 11% of the infants were cared for by their mother, 5% by their father, and 82% of the couples reported that they both took care of the infant at the weekend.

### 2.2 Instruments

#### 2.2.1 Mind‐mindedness

At 4 months parental mind‐mindedness was assessed during a five‐minute free play period at the university research lab. The parent was asked to play with his or her child as naturally as possible. They were allowed to use all available space in the room. Each session began with the infant lying on a couch, with the parent sitting next to the infant. After two‐and‐a‐half minutes of play time without toys, the parent was provided with a box with five age‐appropriate toys to play with. At 12 months, mind‐mindedness was assessed during a 10‐minute free play session. The parent and child were seated on a play mat in the centre of the room with age‐appropriate toys and pillows. After five minutes the toys were replaced with animal‐printed pillows and the parent and child continued their play for another five minutes.

Each comment made by the parent (i.e., each spoken word or sentence) was transcribed and coded by two trained observers using a translated version of the mind‐mindedness coding manual (Meins & Fernyhough, 2015; Zeegers & Colonnesi, 2016). First, each parent comment was classified as either directed at the child's mental state or not (i.e., mind‐related or not mind‐related). The mind‐related comments were categorized according to the specific state the parent referred to. Categories were cognitions (e.g., “you remembered this from the zoo”), likes and dislikes (e.g., “you don't like this rattle”), and emotions (e.g., “you're all excited to play with these toys”). In addition, comments about infants’ epistemic states (i.e., “are you teasing me?”) and comments which were obviously meant to be dialogue said/thought by the infant (e.g., “Daddy, I want you to pick me up”) were also classified as mind related.

Second, each comment in one of the above categories was coded as an appropriate mind‐related comment if one or more of the following conditions were met: (a) the trained coder agreed with the parent's reading of the infant's internal state, (b) the internal state comment linked the infant's current activity with similar events in the past or future (e.g., “do you remember which sound a lion makes from when we went to the zoo?”), or (c) the parent voiced (using the first person) what the child might say if he or she could speak. Comments were classified as non‐attuned when the independent coder believed (a) the parent misread the internal state of the child, or (b) the comment referred to a past or future event that had no obvious relation to the infant's current activity (e.g., “I'm sure you would like to feed the ducks later”).

Interrater agreement was assessed on 90 out of 449 transcripts (20%). First, we assessed the interrater agreement on the number of mind‐related comments using intra‐class correlations (ICC; two‐way random effects model with an absolute agreement definition). Inter‐rater agreement was at 4 months ICC_father_ = .75, ICC_mother_ = .88; at 12 months ICC_father_ = .84, ICC_mother_ = .84. Next, we assessed the interrater agreement on the appropriateness of mind‐related comments by calculating Cohen's kappa. Interrater agreement was at 4 months к_father_ = .82; к_mother_ = .85, at 12 months к_father_ = .92; к_mother_ = .88. Disagreements were resolved by discussion.

#### 2.2.2 Physiological measures

Physiological measures were obtained during baseline (two minutes) and the stranger approach task. The stranger task was designed on the basis of the Laboratory Temperament Assessment Battery (Lab‐TAB; Goldsmith & Rothbart, 1996). The infant was placed in an age‐appropriate seat during the lab visit with the mother. Mothers were seated on a chair behind their infant. During the task, a male stranger approached and talked to the infant for 30 seconds and then picked up and held the infant for 30 seconds. Physiological measures were recorded and analysed with Vsrrp98 software (Molenkamp, 2011). Data acquisition in the programme was performed by a National Instruments NI6224 data acquisition card sampling at a rate of 200S/s per channel. A standard Lead‐II configuration was used to record ECG. In Vsrrp98, R‐waves were identified and adjusted for artefacts. HRV was calculated as the square root of the mean squared differences (RMSSD) of successive normal‐to‐normal (NN) intervals (Task Force of the European Society of Cardiology and the North American Society of Pacing and Electrophysiology, 1996). For baseline HRV, the mean value of HRV during the two‐minute baseline was used. To calculate HRV decline, HRV scores during baseline were subtracted from HRV scores during the stranger approach task. Negative values thus reflected a decrease in HRV during the stranger approach task.

#### 2.2.3 Parenting quality

Parenting quality was assessed at 12 months by scoring parenting behaviour on four scales: responsiveness, intrusiveness (reversed), warmth/affectivity, and negativity (reversed). The scoring was based on the Meso Behavioural Rating System for Families with young children (MeBRF; Mahoney, Coffield, Lewis, & Lashley, 1998; Majdandžić, de Vente, & Bögels, 2016). This coding system requires coders to observe and rate participants’ behaviours, affect, and nonverbal behaviour during structured family interactions (e.g., play or forced‐compliance tasks). Every minute of the free‐play session (five minutes with toys and five minutes without toys) was scored on a 5‐point scale. The scores on the parenting scales were averaged across time intervals, and negative scales (intrusiveness and negativity) were reversed. Subsequently, a composite score of the four scales indicating parenting quality was calculated. The four scales were based on the Erickson Scales and the MeBRF (Erickson, Sroufe, & Egeland, 1985; Mahoney et al., 1998). Responsivity reflected the degree to which the parent responded to the child's initiatives in a sensitive and child‐focused manner. Intrusiveness was indicated by the parent's lack of respect for the child's autonomy by interfering with the child's desires, interests or behaviours. Warmth/affectivity was indicated by parents’ proclivity to show verbal, physical, and facial signs of affection and support towards the infant. Negativity referred to the extent to which parents verbally and nonverbally communicated hostility, rejection, or disapproval towards the child. The coding was done by four couples of raters and a master coder. To prevent rater bias, leading to an overestimation in the correlation between mind‐mindedness and parenting quality, the raters who coded the parenting quality and mind‐mindedness data were different from the raters who coded the data on mind‐mindedness. A total of 15% of the interactions were double coded and the mean inter‐rater agreement across the four couples was *M* ICC = .87 (*SD* = .08, range .76 to .91).

### 2.3 Statistical approach

#### 2.3.1 Missing values

From the participating families, data on mind‐mindedness at 4 months was available for 114 mothers and 110 fathers. At 12 months, data on mind‐mindedness and parenting behaviour was available for 112 mothers and 113 fathers. The scores on mind‐mindedness were missing due to no lab visit at one of the time points, parents speaking a foreign language during the interaction that we did not master (e.g., Japanese), and technical problems. Valid data on baseline HRV were available for 111 and 99 infants at 4 and 12 months, respectively. For HRV decline valid data were available for 97 and 84 infants at 4 and 12 months, respectively. Missing data were due to families not visiting the lab at one of the time points or disturbance due to movement artefacts or technical problems. We conducted *t* tests to examine whether infants with and without missing data at 4 and 12 months had parents with different levels of mind‐mindedness. These *t* tests showed non‐significant results (all *p*‐values > .10). We also coded whether infants cried during the stranger approach, and checked whether the infants who cried were at risk of having a noisy signal due to movement artefacts (chi‐square test of independence). Infants who cried at 4 and 12 months were not more likely to have a noisy signal, 4 months: χ^*2*^ (1) = 0.07, *p* = .795, and 12 months: χ^*2*^ (1) = 0.15, *p* = .700.

#### 2.3.2 Model testing

**Figure 1:**
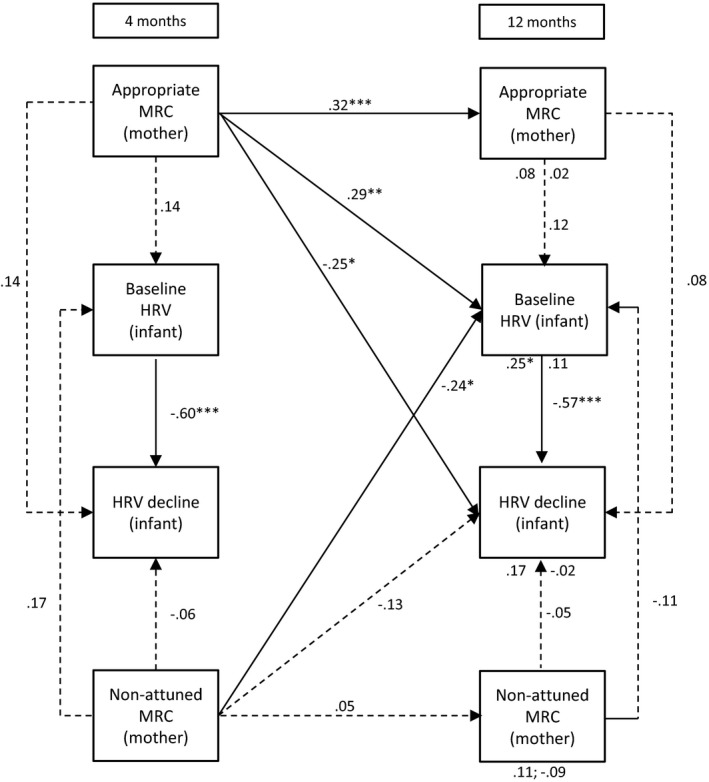
Standardized path coefficients for the relations between mothers’ mind‐mindedness and infant HRV at 4 and 12 months( *Note*: MRC = mind‐related comments; *p < .05; **p < .01; ***p < .001; direct effects of infants’ baseline HRV and HRV decline at 4 months are presented below the variables at 12 months. For instance, the effects of baseline HRV and HRV decline on appropriate MRC were .08 and .02, respectively

**Figure 2:**
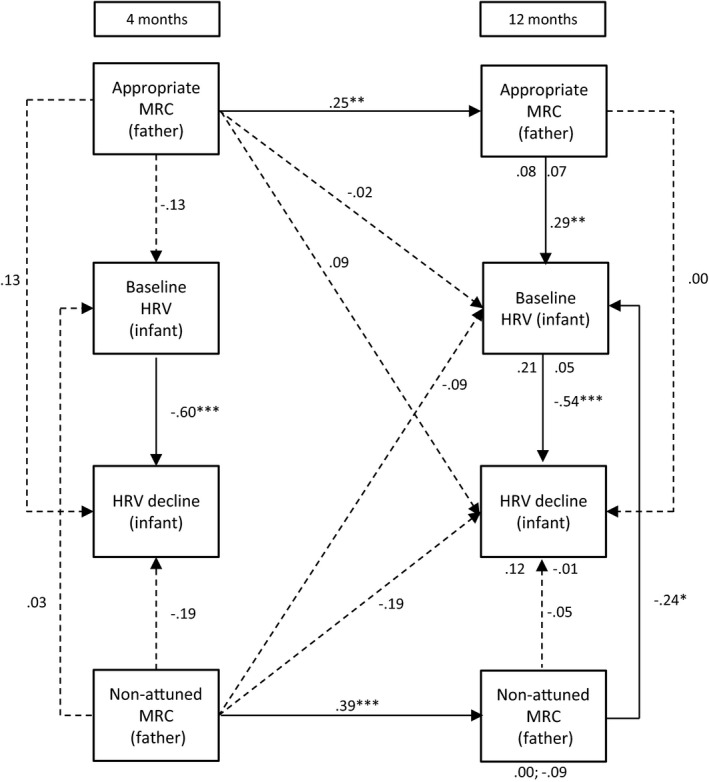
Standardized path coefficients for the relations between fathers’ mind‐mindedness and infant HRV at 4 and 12 months )*Note*: MRC = mind‐related comments; *p < .05; **p < .01; ***p < .001; direct effects of infants’ baseline HRV and HRV decline at 4 months are presented below the variables at 12 months. For instance, the effects of baseline HRV and HRV decline on appropriate MRC were .08 and .02, respectively

**Figure 3:**
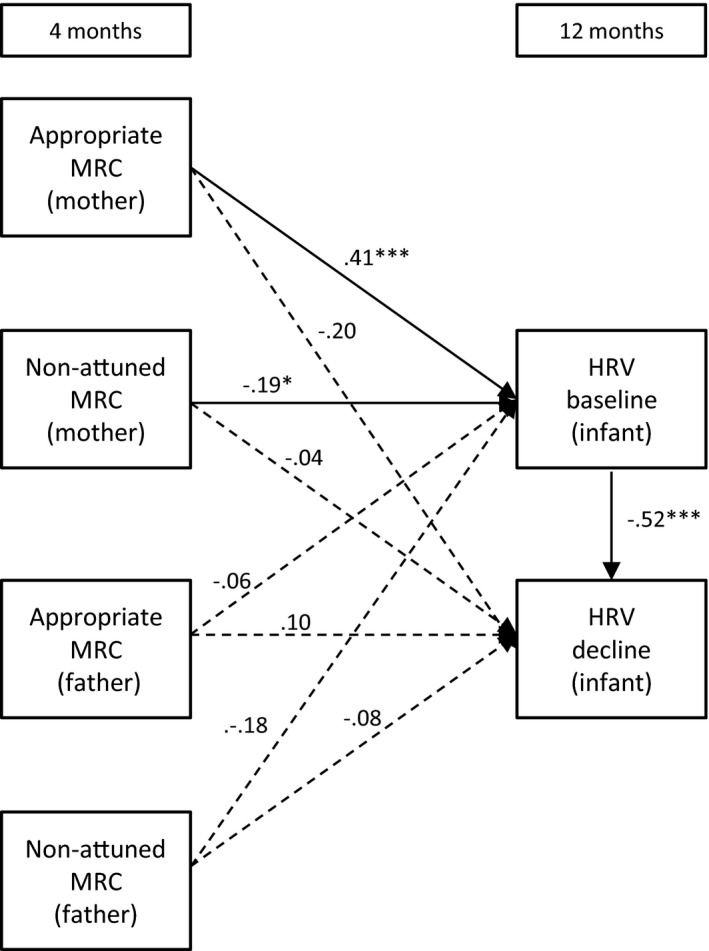
The direct effects of mothers’ and fathers’ mind‐mindedness at 4 months on infant HRV at 12 months *Note*: MRC = mind‐related comments; *p < .05; **p < .01; ***p < .001

**Figure 4:**
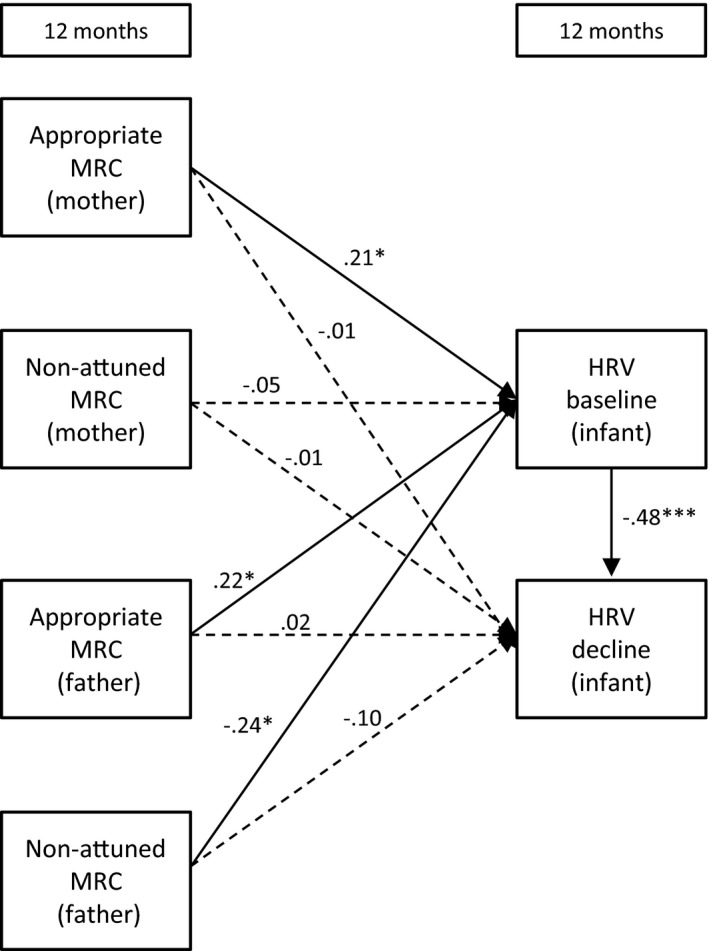
The direct effects of mothers’ and fathers’ mind‐mindedness at 12 months on infant HRV at 12 months *Note*: MRC = mind‐related comments; *p < .05; **p < .01; ***p < .001

**Figure 5:**
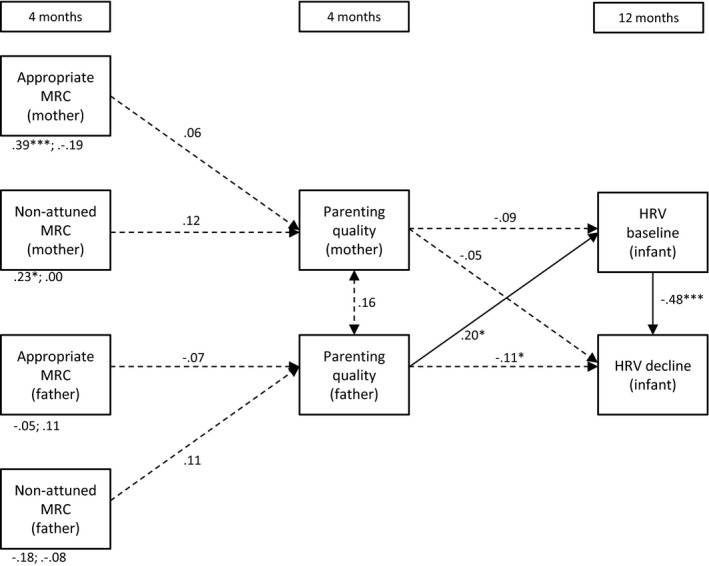
Direct and indirect (via parenting quality) effects of maternal and paternal mind‐mindedness at 4 months on infant HRV at 12 months *Note*: MRC = mind‐related comments; *p < .05; **p < .01; ***p < .001; the direct effects of mind‐mindedness on baseline HRV and HRV decline are displayed below the variables (e.g., .39***; .19 refer to the effects of maternal appropriate mind‐related comments on baseline HRV and HRV decline, respectively)

**Figure 6:**
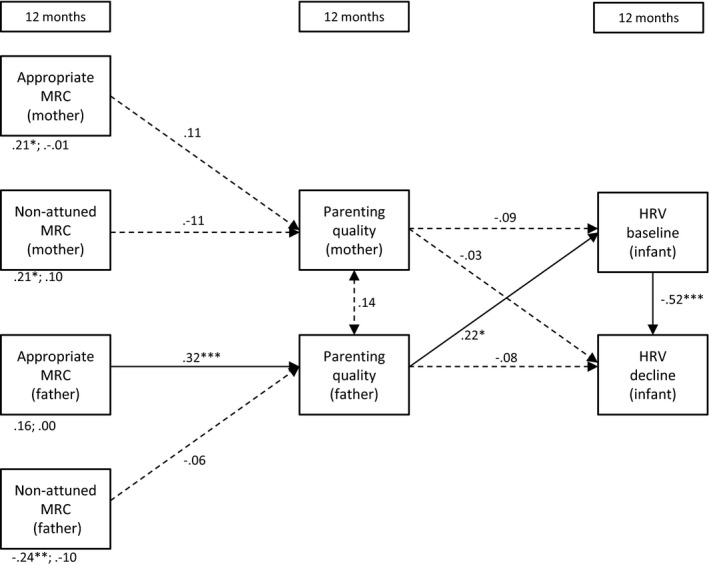
Direct and indirect (via parenting quality) effects of maternal and paternal mind‐mindedness at 12 months on infant HRV at 12 months *Note*: MRC = mind‐related comments; *p < .05; **p < .01; ***p < .001; the direct effects of mind‐mindedness on baseline HRV and HRV decline are displayed below the variables (e.g., .21*; −.01 refer to the effects of maternal appropriate mind‐related comments on baseline HRV and HRV decline, respectively)

Path models were assessed using structural equation modelling (AMOS, 23.0, IBM SPSS, version 22). Missing data were handled using the full information maximum likelihood (FIML) method. The FIML method obtains maximum use of the data, without substituting an actual value for missing data points or deleting cases with missing data points (Kline, 2015). Instead, parameter values are estimated that are most likely to have resulted in the observed sample data. Multiple measures were used to analyse the fit of each model to the observed data: (1) a chi‐square measure of overall goodness of fit, (2) the Comparative Fit Index (CFI), and (3) the root mean square error of approximation (RMSEA). A non‐significant chi‐square coefficient indicates that the hypothesized model does not significantly deviate from the model present in the data. A CFI value close to or greater than .95 warrants that the model is at least a better reflection of the data than a model where all correlations are assumed to be zero. Lastly, the RMSEA coefficient suggests adequate fit when it is close to or less than .08. A value lower than .05 indicates good fit (Kline, 2015). The size and significance of indirect effects were tested with a calculator using a Monte Carlo approach, which is appropriate for structural equation models (Falk & Biesanz, 2016). Below we explain the six models we tested for each of the hypotheses. The models are also visually presented in Figures 1, 2, 3, 4, 5 to 6.

##### Mind‐mindedness predicts infants’ HRV development

The first hypothesis concerns the effects of parents’ mind‐mindedness on the infant's development of HRV from 4 to 12 months. To examine the hypothesis, we tested a path model with all transactional relations between parental mind‐mindedness and infant HRV at 4 and 12 months for mothers and fathers separately (Models 1 and 2). These models enabled us to study whether parental mind‐mindedness predicted infant HRV at 12 months, over and above the effects of infants’ initial HRV status at 4 months. We tested a mother and father model because a path model with all variables (infant, father, mother) included in a single model would be too comprehensive in terms of parameter estimates, considering the amount of participants in this study (Kline, 2015). Model 1 included the transactional relations between infants’ baseline HRV and HRV decline and mothers’ mind‐mindedness at 4 and 12 months. Model 2 tested the same relations for the infant–father dyads. The following pathways were modelled: (a) concurrent relations between baseline HRV and HRV decline during the stranger situation at 4 and 12 months (e.g., Izard et al., 1991; Porter et al., 1995), (b) concurrent relations between appropriate or non‐attuned mind‐related comments and baseline HRV or HRV decline at 4 and 12 months, and (c) longitudinal relations between appropriate or non‐attuned mind‐related comments and baseline HRV or HRV decline at 4 and 12 months. Previous research has shown that appropriate and non‐attuned mind‐related comments are unrelated (e.g., Meins et al., 2003, 2012). Also, in the present study we did not find concurrent and longitudinal correlations between appropriate and non‐attuned comments at 4 and 12 months. Therefore, we did not model concurrent and longitudinal associations between appropriate and non‐attuned mind‐related comments.

##### Mothers’ and fathers’ mind‐mindedness independently predict infant HRV

The second hypothesis involved the independent contributions of mothers and fathers in predicting infant HRV. We tested two models (Models 3 and 4). In Model 3 we examined the direct longitudinal effects of mothers’ and fathers’ mind‐mindedness at 4 months on infant HRV at 12 months. In Model 4 we tested the direct concurrent effects of mothers’ and fathers’ mind‐mindedness at 12 months on infant HRV at 12 months. As both mothers and fathers were included in the same model, we were able to examine whether parents uniquely contribute to infant HRV.

##### Mind‐mindedness predicts infant HRV via parenting behaviour

The third hypothesis focused on whether parents’ mind‐mindedness predicts infant HRV via the quality of parenting behaviour. To examine this hypothesis, we added measures of mothers’ and fathers’ parenting quality at 4 and 12 months to the above‐mentioned models (in Models 5 and 6). In Model 5 we tested the indirect effects of mothers’ and fathers’ mind‐mindedness at 4 months via parenting quality at 4 months on infant HRV at 12 months. In Model 6 we tested the indirect effects of mothers’ and fathers’ mind‐mindedness at 12 months via parenting quality at 12 months on infant HRV at 12 months. For both models we first tested whether a full mediation model would fit the data. Subsequently we examined whether the model fit improved after adding the direct effects of mind‐mindedness to the model (i.e., a partial mediation model).

#### 2.3.3 Data distribution

Paternal appropriate and non‐attuned mind‐mindedness at 4 months, maternal non‐attuned mind‐mindedness at 12 months and baseline HRV at 4 months had outliers (z > 3.29), which may exert a disproportionate influence on the results. We assigned the outliers with scores one unit larger than the next most extreme score (see Tabachnick & Fidell, 2013, p. 11). We reran the analyses with and without the altered variables. Results were very similar, indicating that the outliers did not impact the outcomes. We present the results with the unaltered variables. The distribution of non‐attuned mind‐mindedness was skewed because approximately 45% of the parents did not make any non‐attuned comments. Variables were (log) transformed. The analyses with and without the transformed variables led to similar results. The untransformed variables were used in the final analyses.

## 3 RESULTS

Descriptive statistics for the infant and parent variables are reported in Table 1. Overall, infants showed a decrease in HRV during the stranger task at 4 (*M* = −1.16, *SD* = 10.01) and 12 months (*M* = −1.92, *SD* = 15.30). The mean proportions of appropriate mind‐related comments at 4 months produced by mothers and fathers were 7% and 8%, respectively. The mean proportion of appropriate mind‐related comments at 12 months decreased slightly to 6% for both mothers and fathers. Non‐attuned mind‐related comments were made less often: at both time points means were 1% for both mothers and fathers.

**Table 1:** Descriptive statistics for the variables included in the path analyses

		*N*	*M* (*SD*)	Range
*Infant*				
HRV baseline	4 months	111	16.57 (9.85)	6.16; 50.52
	12 months	99	29.88 (16.75)	7.84; 82.04
HRV decline	4 months	97	−1.16 (10.01)	−36.98; 29.84
	12 months	84	−1.92 (15.30)	−63.57; 34.59
*Mother*				
Mind‐mindedness^a^	Appropriate MRC 4	114	0.07 (0.05)	0; .21
	Non‐attuned MRC 4	114	0.01 (0.01)	0; .05
	Appropriate MRC 12	112	0.06 (0.03)	0; .15
	Non‐attuned MRC 12	112	0.01 (0.01)	0; .03
Parenting quality	12 months	116	4.22 (0.18)	3.50; 4.59
*Father*				
Mind‐mindedness	Appropriate MRC 4	110	0.08 (0.06)	0; .26
	Non‐attuned MRC 4	110	0.01 (0.02)	0; .09
	Appropriate MRC 12	113	0.06 (0.03)	0; .15
	Non‐attuned MRC 12	113	0.01 (0.01)	0; .05
Parenting quality	12 months	116	4.14 (0.19)	3.53; 4.70

Note

MRC = mind‐related comments.

a Descriptives for mind‐related comments are displayed in percentages.

Correlations between the mind‐mindedness variables and parenting quality, as well as the concordance within couples are presented in Table 2. For both mothers and fathers, appropriate mind‐related comments were stable from 4 to 12 months, *r*_mothers_(105) = .33, *p* = .001; *r*_fathers_(102) = .24, *p* = .016. Non‐attuned mind‐related comments showed stability only for fathers, *r*_fathers_(102) = .42, *p* < .001; *r*_mothers_(102) = .03, *p* = .79. There was concordance in mothers’ and fathers’ appropriate mind‐related comments at 12 months, *r*(109) = .22, *p* = .018. Table 3 presents the correlations between the predictor and outcome variables.

**Table 2:** Pearson's R correlations (*N*)^a^ between appropriate and non‐attuned mind‐mindedness and parenting quality for mothers and fathers at 4 and 12 months^b^

		1	2	3	4	5
4 months	1. Appropriate MRC	.**06 (104)**	.12 (110)	.24* (102)	.12 (102)	−.01 (103)
	2. Non‐attuned MRC	−.10 (114)	**.12 (014)**	.13 (102)	.42*** (102)	.09 (103)
12 months	3. Appropriate MRC	.33** (105)	.01 (105)	**.22** * **(109)**	.15 (113)	. 31** (108)
	4. Non‐attuned MRC	.00 (105)	.01 (105)	−.11 (112)	**.04 (109)**	−.02 (108)
	5. Parenting quality	.01 (106)	.00 (106)	.13 (106)	−.13 (106)	**.16 (116)**

Note

*p < .05; **p < .01; ***p < .001; MRC = mind‐related comments.

a The sample size (*N*) for the particular correlation is reported between parentheses.

b Correlations between the paternal variables are displayed above the diagonal, and correlations between the maternal variables are presented below the diagonal. The diagonal correlations display the concordance between mothers and fathers.

**Table 3:** Pearson's R correlations (*N*)^a^ among the HRV measures and between infant HRV at baseline and maternal and paternal mind‐mindedness at 4 and 12 months

	HRV bas 4	HRV dcl 4	HRV bas 12	HRV dcl 12
HRV baseline 4		−.60***	.20	.06
HRV decline 4			−.07	−.02
HRV baseline 12				−.48***
*Mothers*				
Appropriate MRC 4	.17	−.19	.39***	−.32**
Non‐attuned MRC 4	.12	.02	−.26**	.02
Appropriate MRC 12	.10	.00	.28**	−.14
Non‐attuned MRC 12	.18	−.12	−.14	.07
Parenting quality 12	−.06	.16	.03	−.03
*Fathers*				
Appropriate MRC 4	−.16	.16	−.02	.06
Non‐attuned MRC 4	.06	−.19	−.18	−.03
Appropriate MRC 12	.00	.04	.23*	−.06
Non‐attuned MRC 12	.05	−.14	−.24*	.23*
Parenting quality 12	.02	.01	.28	−.03

Note

*p < .05; **p < .01; ***p < .001; HRV B = infants’ resting HRV (baseline),

HRV dcl = infants’ HRV decline during the stranger task (compared to baseline),

MRC = mind‐related comments.

### 3.1 Path analyses

#### 3.1.1 Model 1: Maternal mind‐mindedness and infant HRV

Figure 1 displays Model 1. The model closely fits the observed data, χ^*2*^(3) = 1.35, *p* = .718, CFI = 1, RMSEA = .00. Mothers’ appropriate, but not non‐attuned mind‐related, comments were moderately stable over time, β_appropriate_ = .32, *SE* = .09, *p* < .001; β_non‐attuned_ = .05, *SE* = .10, *p* = .642. Mothers’ appropriate mind‐related comments at 4 months predicted higher levels of baseline HRV at 12 months, β = .29, *SE* = .10, *p* = .004, as well as greater HRV decline during the stranger task, β = −.25, *SE* = .11, *p* = .021. Non‐attuned comments at 4 months predicted lower levels of baseline HRV at 12 months, β = −.24, *SE* = .09, *p* = .011. Infants’ baseline HRV at 4 months did not predict mind‐mindedness at 12 months. Mothers’ mind‐mindedness at 12 months was unrelated to infant HRV at 12 months. Infants with higher HRV baseline levels showed a stronger decline in HRV levels during the stranger approach at 4 months, β = −.60, *SE* = .11, *p* < .001 and at 12 months β = −.57, *SE* = .11, *p* < .001, than infants with lower HRV baseline. Infants’ baseline HRV at 4 months was positively related to baseline HRV at 12 months, β = .25, *SE* = .12, *p* = .040.

#### 3.1.2 Model 2: Paternal mind‐mindedness and infant HRV

Figure 2 displays Model 2. The model closely fits the observed data, χ^*2*^(3) = 4.16, *p* = .527, CFI = 1, RMSEA = .00. Fathers’ appropriate and non‐attuned mind‐related comments were stable over time, β_appropriate_ = .25, *SE* = .10, *p* = .010; β_non‐attuned_ = .39, *SE* = .09, *p* < .001. Fathers’ appropriate mind‐related comments at 12 months were related to higher infant baseline HRV at 12 months, β = .29, *SE* = .10, *p* = .002, whereas fathers’ non‐attuned comments at 12 months were related to lower baseline HRV at 12 months, β = −.23, *SE* = .10, *p* = .022. Infant HRV at 4 months did not predict paternal mind‐mindedness at 12 months.

#### 3.1.3 Model 3: Independent effects of maternal and paternal mind‐mindedness at 4 months

Figure 3 displays Model 3, including mothers’ and fathers’ mind‐mindedness at 4 months predicting infant HRV at 12 months. Because all possible paths were examined in this model, the model is fully saturated, and fit indices are not informative (i.e., there is a perfect fit). Mothers’ appropriate mind‐related comments at 4 months predicted higher levels of baseline HRV at 12 months, β = .41, *SE* = .09, *p* < .001, whereas mothers’ non‐attuned mind‐related comments predicted lower baseline HRV at 12 months, β = .19, *SE* = .09, *p* = .048. Maternal appropriate comments no longer predicted greater HRV decline, β = .20, *SE* = .11, *p* = .070.

#### 3.1.4 Model 4: Independent effects of maternal and paternal mind‐mindedness at 12 months

Figure 4 displays Model 4, including mothers’ and fathers’ mind‐mindedness at 12 months predicting infant HRV at 12 months. Again, the fit indices were not informative because all possible paths were examined. Mothers’ appropriate mind‐related comments at 12 months predicted higher levels of baseline HRV at 12 months, β = .21, *SE* = .10, *p* = .031. Fathers’ appropriate mind‐related comments at 12 months predicted higher levels of baseline HRV at 12 months, β = .22, *SE* = .10, *p* = .025, whereas fathers’ non‐attuned mind‐related comments predicted lower baseline HRV at 12 months, β = −.24, *SE* = .10, *p* = .011.

#### 3.1.5 Model 5: Mind‐mindedness predicts infant HRV via parenting quality at 4 months

We first tested whether mind‐mindedness at 4 months was associated with parenting quality at the same age, to examine whether this, in turn, predicted infants’ HRV levels at 12 months (i.e., a full mediation model). This model showed a poor fit to the observed data, χ^*2*^(12) = 40.67, *p* < .001, CFI = .37, RMSEA = .14. We then added the direct effects of mind‐mindedness (4 months) on infant HRV at 12 months, χ^*2*^(4) = 8.66, *p* = .070, CFI = .89, RMSEA = .09. A chi‐square difference test of the full mediation model against the partial mediation model was significant, χ^*2*^(8) = 32.01, *p* < .001, indicating that the partial mediation model showed a better fit to the observed data than the full mediation model (Kline, 2015). The estimates of the partial mediation model are presented in Figure 5. Mind‐mindedness at 4 months was unrelated to parenting quality at 4 months. Mothers’ appropriate mind‐related comments at 4 months predicted higher levels of baseline HRV at 12 months, β = .41, *SE* = .09, *p* < .001, whereas mothers’ non‐attuned mind‐related comments predicted lower baseline HRV at 12 months, β = .19, *SE* = .09, *p* = .048. When fathers scored higher on parenting quality at 4 months, infants had higher levels of baseline HRV at 12 months, β = .20, *SE* = .09, *p* = .024.

#### 3.1.6 Model 6: Mind‐mindedness predicts infant HRV via parenting quality at 12 months

We first examined whether mind‐mindedness at 12 months predicted parenting quality at 12 months, to examine whether this, in turn, predicted infants’ HRV levels at 12 months (i.e., a full mediation model). This model showed a satisfactory fit to the observed data, χ^*2*^(12) = 18.60, *p* < .001, CFI = .85, RMSEA = .07. We added to the model the direct effects of mind‐mindedness (12 months) on infant HRV at 12 months, χ^*2*^(4) = 2.14, *p* = .710, CFI = 1, RMSEA = .00. A chi‐square difference test of the full mediation model against the partial mediation model was significant, χ^*2*^(8) = 16.46, *p* = .036. This indicates that the partial mediation model showed a better fit to the observed data than the full mediation model (Kline, 2015). The estimates of the partial mediation model are presented in Figure 6.

Paternal appropriate mind‐related comments were positively related to fathers’ parenting quality. In turn, fathers who showed higher levels of parenting quality at 12 months were more likely to have infants with higher baseline levels. The indirect effect of paternal appropriate mind‐related comments on infant HRV via parenting quality was significant, β = .08, CI 95% [0.01,0.18]. The direct effect of fathers’ appropriate mind‐related comments on infant HRV was no longer significant in this model. Fathers who made non‐attuned mind‐related comments were more likely to have infants with lower HRV baseline levels, β = .24, *SE* = .09, *p* = .009. Mothers’ appropriate mind‐related comments positively related to infant baseline HRV at 12 months, β = .21, *SE* = .10, *p* = .031. Parenting quality of mothers was unrelated to maternal mind‐mindedness and infant HRV.

#### 3.1.7 Robustness analyses

Prior to the analyses, we checked whether any of the predictor variables were related to infant gender and the level of education of the parents. Since mothers with a higher education level made fewer non‐attuned comments at 12 months, *r*(109) = −.20, *p* = .036, we reran the two maternal path analyses including mothers’ educational level as a covariate. The outcomes of these analyses were essentially similar to the outcomes presented in Figures 1 and 2.

## 4 DISCUSSION

The present study tested three hypotheses: (a) parents’ mind‐mindedness at 4 and 12 months predicts infant HRV levels at 12 months over and above infants’ initial HRV levels at 4 months, (b) mothers’ and fathers’ mind‐mindedness independently predict infant HRV, and (c) the effects of mind‐mindedness on infant HRV (partially) operate through parenting behaviour. With regard to the first hypothesis, we found that mothers’ higher proportions of appropriate mind‐related comments during interactions with their infants at 4 and 12 months predicted infants’ higher baseline HRV at 12 months, whereas higher proportions of non‐attuned comments at 4 months predicted lower baseline HRV at 12 months. Furthermore, we observed a larger HRV decline in infants during the stranger task at 12 months when mothers made more frequent appropriate mind‐related comments at 4 months. For fathers, higher proportions of appropriate mind‐related comments at 12 months were associated with infants’ higher baseline HRV at 12 months, whereas higher proportions of non‐attuned comments at 12 months were associated with lower baseline HRV at 12 months. Paternal non‐attuned comments at 12 months were related to less HRV decline during the stranger task. Infants’ baseline HRV and HRV decline at 4 months did not predict mothers’ and fathers’ mind‐mindedness at 12 months.

With regard to the second hypothesis, we found concordance within couples regarding appropriate mind‐related comments at 12 months (but not at 4 months). Parents thus start to become more alike in terms of their mind‐mindedness. It may be that parents influence each other's mentalizing stance and mind‐related speech throughout the first year of the infant's life. It could also be that the child's individual characteristics increasingly evoke higher or lower mind‐mindedness in both parents, and thus couples’ mind‐mindedness starts to correlate at a later age (at least after 4 months). Despite the correlation between paternal and maternal appropriate mind‐related speech at 12 months, mothers’ and fathers’ mind‐mindedness independently predicted infant HRV. With regard to the third hypothesis, paternal, but not maternal, mind‐mindedness showed an indirect effect on infant HRV via parenting quality. Fathers’ appropriate mind‐related comments at 12 months were positively related to parenting quality, which in turn positively related to infant baseline HRV.

We tested physiological emotion regulation capacity in two situations, during a baseline period and a potentially stressful situation. Infants’ baseline HRV was found to be somewhat stable over time, which is in line with the majority of the studies conducted previously (e.g., Bar‐Haim et al., 2000; Bornstein & Suess, 2000). The extent to which HRV decreased from baseline during the stranger task was not stable over time. These findings support the conception that baseline HRV reflects the infant's general capacity and flexibility to regulate arousal, while a decline in HRV reflects how the parasympathetic system reacts to a specific challenge (Appelhans & Luecken, 2006; Porges, 2011). Although the stranger approach task was similar during the two measurements, it may be processed and appraised differently at different ages. At 4 months, infants are able to recognize familiar and unfamiliar faces, but they do not seem to associate the recognition of particular individuals with the (helpful or disruptive) responses of these individuals, as they do when they are 12 months (Bushnell, 1998). Unfamiliarity with an individual may thus not be processed and perceived as equally “threatening” at 4 and 12 months of age. Hence, parasympathetic reactivity during a stranger task may be less stable over the infant's first year compared to parasympathetic activity during rest.

In line with prior research, appropriate mind‐related comments were stable over time in both mothers and fathers (e.g., Kirk et al., 2015). Non‐attuned comments were only stable for fathers. These comments occurred rather infrequently; 1% of the total amount of comments for both mothers and fathers. Visual inspection of the data, however, showed that for fathers there was a substantial group showing three or more non‐attuned comments (16% at 4 months), whereas this group was much smaller for mothers (8% at 4 months). Similar percentages were found at 12 months. Thus, when parents made non‐attuned comments, fathers expressed higher frequencies of non‐attuned comments compared to mothers. The relatively small range and variation in maternal non‐attuned comments may explain why no stability was found for mothers’ non‐attuned comments from 4 to 12 months.

The outcome that both mothers’ and fathers’ appropriate and non‐attuned mind‐related comments were positively and negatively linked to infants’ baseline HRV supports the idea that caregivers shape the infant's developing emotion regulation capacity (Eisenberg, Cumberland, & Spinrad, 1998). This notion that mind‐mindedness is an important and unique facilitator of emotion regulation is further strengthened by the fact that mind‐mindedness predicted infant HRV over and above parenting quality. According to attachment theory, emotion regulation skill is a product of the quality of an enduring infant–parent relationship (Fonagy et al., 2004). In that sense, the present results also connect to previous findings identifying mind‐mindedness as a unique predictor of infants’ attachment security (Meins et al., 2001, 2012; Zeegers et al., 2017).

Appropriate and non‐attuned comments made independent contributions to the prediction of emotion regulation, underlining the notion that the two indices represent two distinct dimensions of mind‐mindedness (Meins, 2013): one dimension that reflects whether the parent is inclined to represent the infant's mind explicitly and accurately, and one that reflects whether the parent attributes mental states to the infant that are not matched to the infant's presumed state (Meins et al., 2012). The tendency to make frequent and appropriate comments on infants’ mental processes is thought to represent parents’ more general sensitive attitude, in contrast to non‐attuned comments (Meins et al., 2012; Zeegers et al., 2017). Meins et al. have argued that parents may show sensitive behaviours despite the fact that their discourse shows a lack of attunement to the infant's internal state. Non‐attuned comments may be indicative of more subtle failures of attunement, different from the more obvious insensitive behaviours such as frightening, hostile, or intrusive behaviours. This nuance might be particularly important for understanding how parent–infant interactions contribute to differences in socio‐emotional development in normative samples (such as in the present study). Parents without mental health problems are less likely to display severe insensitive behaviours, but a lack of fine‐grained attunement may still be present (Wan & Green, 2009). The more subtle failures of attunement may be observed better through analysing the content of their speech than their behaviour. The combination of appropriate and non‐attuned mind‐related comments has been successfully used to differentiate parents of children with different insecure attachment classifications (Meins et al., 2012). The results of the present study are in accordance with these prior results, indicating that appropriate and non‐attuned mind‐related comments uniquely contribute to the prediction of infants’ emotional development.

We found different (independent) effects of mind‐mindedness for mothers and fathers. First, fathers’ appropriate and non‐attuned mind‐related speech related only concurrently to infant HRV at 12 months, as opposed to the concurrent and predictive effect of mothers’ mind‐mindedness. Given the fact that there was considerable stability in fathers’ mind‐related comments, it was surprising that only the 12‐month variable was related to infant HRV. Because the path model simultaneously estimated the path coefficients for all relations in the model, we checked the raw correlations. These also showed no association between fathers’ mind‐mindedness at 4 months and infants’ later HRV levels. The magnitude of the paternal and maternal path coefficients were very similar, with appropriate comments showing larger effects than non‐attuned comments.

Second, the effects of parents’ mind‐mindedness on HRV decline during the stranger approach also differed between mothers and fathers. Only for fathers, non‐attuned mind‐related comments at 12 months were associated with less HRV decline during the stranger task. Thus, when fathers tended to misinterpret the infant's state of mind, or project their own state of mind onto that of the child, their infants showed less active emotion regulation during the stranger situation. On the other hand, only for mothers, appropriate mind‐related comments at 4 months predicted stronger HRV decline. Mothers’ appropriate mindreading early in life may thus stimulate infants’ parasympathetic system to become more actively involved in regulation arousal during stressful and unfamiliar social situations. Third, only fathers’ mind‐mindedness at 12 months was related to infants’ HRV directly and indirectly via parenting quality at 12 months. Fathers’ tendency to form representations of the infant's mind thus seemed to connect to responsive, non‐intrusive, affective parenting behaviour, which in turn explained part of the variation in infants’ emotion regulation.

Altogether the findings described above are in line with proposed paternal and maternal differences regarding child development and the proposed unique role of fathers in the prediction of children's emotional development (e.g., Bögels & Perotti, 2011; Lamb, 2000; Paquette, 2004). From an evolutionary perspective, fathers are considered to be more inclined to challenge their children, stimulate risk taking, and more often engage in playful interactions with their children, whereas mothers tend to have a more caring and nurturing parenting role (Möller, Majdandžić, de Vente, & Bögels, 2013). These roles imply that parents may impact their child's development differently at different age points. Four‐month‐old infants have fewer key skills (e.g., motor and communication skills) that allow them to intentionally use parents to regulate their emotional states (Sroufe, 1997). The development of the young infant may therefore be particularly influenced by the extent to which parents adequately read the infant's subtle cues and provide a caring response to these cues. On the other hand, the positive impact of parents who are challenging, yet in an attuned manner, may become more evident at a later age, when infants start to experience more efficacy in modulating their own and others’ behaviours (Majdandžić et al., 2016).

Most of the parents in this study worked at least three days per week. More fathers worked full‐time than mothers. In the Netherlands, female employees are entitled to at least three to four months maternity leave, whereas male employees are entitled to up to two days of paternity leave after their partners have given birth. Mothers are therefore likely to spend more time with their infant in the first months. In the present study, mothers took care of their infant during weekdays more often than fathers (40% versus 11%). Getting to understand and respond appropriately to the emotional arousal of an infant may require time to develop, which mothers in the present sample may have had more than fathers. Furthermore, nursing mothers engage in an interaction that is critical in soothing arousal during the first months. Differences in the nature and quantity of infant–father and infant–mother interactions during the infant's first months may thus also explain the earlier onset of the maternal effects.

Parenting quality at 4 months did not relate to appropriate mind‐related comments at 4 months, whereas fathers’ parenting quality at 12 months did correlate with appropriate mind‐related comments at 12 months. Thus, at 4 months, parents’ mind‐minded stance is not (yet) guiding their parenting behaviours during interaction. One explanation is that at 4 months first‐time parents are very new to parenting in general and they are just learning about the mind of their infant. For instance, parents could just be beginning to understand that their 4‐month‐old is highly sensitive to external stimuli and easily over‐aroused. Their behaviour may not yet be guided by this kind of early “mental sensitivity”. Another explanation is that at 4 months, parenting is more non‐verbal than at 12 months (e.g., comforting the baby when upset), and not yet guided by verbal comments about their mental states (e.g., “you’re upset and you need a hug”). At 12 months, fathers who tend to be attuned to their infant's state of mind also tend to be more responsive and warm, and less intrusive and negative. This suggests that the fathers representational mindset and their actual behaviour become synchronized throughout the first year of the infant's life.

That the synchronization of mind‐mindedness and parenting behaviour was found in fathers but not in mothers is surprising considering a meta‐analysis and review that suggest that mothers’ appropriate mind‐related commenting is related to sensitive parenting typically assessed with the Ainsworth scales, Maternal Behavior Q‐sort or the Emotional Availability Scales (McMahon & Bernier, 2017; Zeegers et al., 2017). The measure that we used in the present study, however, entailed a composite of multiple parenting behaviours (i.e., responsivity, intrusiveness, warmth and negativity). The responsivity measure reflected the degree to which the parent responded to the child's initiatives in a sensitive and child‐focused manner, which is similar to parental sensitivity. We therefore checked post hoc the correlation between responsivity and maternal and paternal appropriate mind‐mindedness. For fathers the correlation was significant, *r* = .23, *p* = .016. For mothers, the correlation was in the positive direction, but did not reach significance, *r* = .15, *p* = .115. Although meta‐analytic evidence shows that the pooled correlation between appropriate mind‐related comments and sensitivity is significant, the range of correlations among studies varies greatly, from *r* =. 14 (Demers, Bernier, Tarabulsy, & Provost, 2010) to *r* = .41(Rosenblum, McDonough, Sameroff, & Muzik, 2008; see Zeegers et al., 2017 online supplementary material). Whether these inconsistencies in findings are explained by, for instance, sample or methodological differences between studies is not clear yet, as the moderator analyses in Zeegers et al. (2017) did not point out clear moderators of this relation. The reasons for the varying correlations should therefore be further addressed in future research. Very few studies have investigated the (longitudinal) relation between mind‐mindedness and parenting of fathers versus mothers, and future research is needed for a better understanding of differences in (the development of) mind‐mindedness and parenting quality in fathers and mothers.

Infants’ HRV at 4 months did not predict parents’ mind‐mindedness. As we outlined in the Introduction, infants’ HRV at 4 months primarily seems to reflect their responsiveness to, or engagement with, the environment, in both positive and negative contexts (Beauchaine, 2001). The present results suggest that this type of early responsiveness does not shape whether and how accurately parents represent the mind of their child. This is in line with the prior finding that mind‐mindedness is unrelated to infant temperament (Meins et al., 2011). However, we should not rule out the possibility that the relation between mind‐mindedness and infant emotion regulation is transactional or bidirectional, as we found concurrent, and not predictive, associations between the paternal mind‐mindedness indices and infant HRV. Thus, it seems realistic to expect that if parents’ mind‐mindedness is shaped by the child's characteristics, this is a gradual process occurring over a longer time span.

### 4.1 Limitations and future directions

The current study has several limitations. First, although parents’ mind‐related comments were not predicted by infants’ emotion regulation, the study was not experimental, precluding conclusions about causality. Second, the sample consisted of a community sample of mothers and fathers with high socioeconomic status, and hence the results cannot be generalized to samples from a different socioeconomic background. Third, in the present study we did not control for infants’ respiration. Changes in respiratory patterns may influence HRV independent of cardiac autonomic activity (Quintana & Heathers, 2014). However, we used a short‐term time‐domain measure of HRV (RMSSD) in the present study which has been shown not to be influenced by respiration rate (Schipke, Arnold, & Pelzer, 1999).

The measure of parenting quality we used in this study is rather broad and it seems interesting to consider that, maybe in hindsight, it is too coarse‐grained to grasp the link between mothers’ early (4‐month) mind‐mindedness and the infant's development of emotion regulation. The body‐based interactive processes between the parent and the infant play an important role in understanding the effects of parents’ mentalizing stance, especially in the case of very young infants. Parental Embodied Mentalization (PEM) is a construct developed to assess parental mentalization at the nonverbal and primarily unconscious level (Shai & Belsky, 2011). PEM refers to the parental capacity to implicitly conceive, comprehend, and extrapolate the infant's mental states from the infant's whole body kinaesthetic expressions and adjust their own kinaesthetic patterns accordingly (Shai & Belsky, 2011). The combination of parents’ implicit (PEM) and explicit (mind‐mindedness) mentalization may shed further light on infant–parent processes that shape the infant's emotion regulation.

Next to understanding how mind‐mindedness operates indirectly, it seems relevant to gain more insight into the direct effects of mind‐mindedness. For instance, is there an underlying shared genetic trait that explains the direct effects of mind‐mindedness on HRV? Furthermore, little is known about when the effect of mental state language on the infant's emotional development becomes apparent. Is exposure to mental state language already important even before the infant is able to fully decode parental speech? It may be that parental language has a stronger impact during the pre‐verbal stage than appears at first sight. Previous studies have shown that from the age of 6 months infants show signs of understanding adult speech, and they begin to imitate speech as early as in the second half‐year of life. Also, the ability to symbolize spoken language gradually starts to unfold before they turn 1 year old (e.g., Bates, 2014). This means that even before infants can speak, they begin to appreciate that reality can be represented through the use of abstract concepts (Piaget, 1950). Mind‐related speech, which is the parent's active validation and symbolization of the infant's internal and physiological state, may therefore already influence the infant's organization of emotional arousal through the symbolizing abilities of infants at a very young age.

A next step in this line of research is to address whether the effects of mind‐mindedness extend to emotion regulation capacity in childhood, and to other facets of emotion regulation. For instance, is mind‐mindedness also predictive of children's cognitive and behavioural strategies to cope with emotions? Particularly from the time children become able to understand and produce spoken language, the impact of mind‐mindedness may become more evident. The parent's tendency to use mind‐related speech while interacting with the child is thought to offer children verbal tools with which to progress from being externally regulated to self‐regulated (Bernier, Carlson, & Whipple, 2010). First support for this notion comes from a study reporting that children whose mothers were more mind‐minded when they were 12 months old performed better on working memory at 18 months, and were inclined to perform better on both conflict and impulse control at 26 months (Bernier et al., 2010). Second support for this notion comes from a study reporting that children whose fathers were more mind‐minded when they were 18 months old performed better at an inhibitory control task (Gagné et al., 2018).

Lastly, another topic that has been left unstudied are the independent effects of parents’ mind‐mindedness. We explored the effects of paternal and maternal mind‐mindedness in isolation. However, parents form a dynamic system and may strengthen or compensate for each other's influence on the infant's development. For instance, having a mother who is unstable in interpreting the infant's mind correctly (i.e., high in appropriate and non‐attuned mind‐related comments) in combination with a father who is not inclined to interpret the infant's mind (i.e., low in appropriate and non‐attuned mind‐related comments) may result in more regulation problems compared to an infant that has either one of these parents in combination with a highly attuned parent (i.e., high in appropriate and low in non‐attuned mind‐related comments). This type of research would help to gain insight into familial risk factors.

### 4.2 Conclusion

Both parents’ appropriate and non‐attuned mind‐related commenting linked to the developing status of infants’ HRV levels, although the impact of mothers’ mind‐mindedness may show an earlier onset. The effects of parental mind‐mindedness held over and above the effects of parenting quality. The present study provides initial evidence that mothers’ and fathers’ tendency to make sense of their infant's mind in an attuned manner influences the development of infants’ emotion regulation capacity throughout the first year of life.
